# Induction of E-cadherin^+^ human amniotic fluid cell differentiation into oocyte-like cells via culture in medium supplemented with follicular fluid

**DOI:** 10.3892/mmr.2014.2199

**Published:** 2014-04-29

**Authors:** TE LIU, YONGYI HUANG, YANZHEN BU, YANHUI ZHAO, GANG ZOU, ZHIXUE LIU

**Affiliations:** 1 Shanghai Geriatric Institute of Chinese Medicine, Longhua Hospital, Shanghai University of Traditional Chinese Medicine, Shanghai 200031, P.R. China; 2 School of Life Science and Technology, Tongji University, Shanghai 200092, P.R. China; 3 College of Life Science, Henan Normal University, Xinxiang 453007, P.R. China; 4 Department of Oral and Craniofacial Science, Ninth People’s Hospital, Shanghai Jiao Tong University School of Medicine, Shanghai 200011, P.R. China; 5 Shanghai First Maternity and Infant Hospital, Tongji University School of Medicine, Shanghai 200040, P.R. China

**Keywords:** HuAFCs, E-cadherin, DAZL, follicular fluid, oocyte-like cells, siRNA

## Abstract

Pluripotent human amniotic fluid cells (HuAFCs) can differentiate into various types of somatic cell *in vitro*. However, their differentiation into oocyte-like cells has never been described to the best of our knowledge. In the present study, differentiation of E-cadherin^+^ and E-cadherin^−^ HuAFC sub-populations into oocyte-like cells was induced via culture in medium containing bovine follicular fluid and β-mercaptoethanol. The E-cadherin^+^ HuAFCs expressed DAZL highly. Post-induction, cells with an oocyte-like phenotype were found among the E-cadherin^+^ HuAFCs, expressing markers specific to germ cells and oocytes (VASA, ZP3 and GDF9) and meiosis (DMC1 and SCP3). When specific small interfering RNA (siRNA) was used to suppress E-cadherin in the E-cadherin^+^ HuAFCs, the levels of DAZL expression were reduced. Post-induction, the morphology of the siRNA-E-cadherin HuAFCs was poorer and the expression levels of germ cell-specific markers were lower compared with those of the siRNA-mock HuAFCs. Therefore, E-cadherin^+^ HuAFCs could be more easily induced to differentiate into oocyte-like cells by bovine follicular fluid and β-mercaptoethanol. In addition, the E-cadherin^+^ HuAFCs exhibited potential characteristics of DAZL protein expression, and thus it was conjectured that bovine follicular fluid acts on DAZL protein and promotes E-cadherin^+^ HuAFC differentiation into oocyte-like cells.

## Introduction

The number of patients worldwide with infertility is increasing every year (1,2). Oocyte defects account for a large proportion of cases of female infertility (2). However, the factors and conditions regulating the development and maturation of oocytes remain unclear. The fact that stem cells can be induced to differentiate into oocytes *in vitro* is of great significance for the treatment of infertility (1–4). Anderson *et al* (5) demonstrated that STRA8 regulated meiotic initiation during spermatogenesis and oogenesis in mice, and meiosis was initiated in germ lines following retinoic acid induction of STRA8 expression. Furthermore, the early mitotic development of germ cells appeared to be undisturbed when the STRA8 gene was knocked out in juvenile C57BL/6 male mice (5). Yu *et al* (6) then demonstrated that via transfection of DAZL, a germ cell-specific RNA-binding protein, mESCs could be induced to differentiate into germ cells directly *in vitro* without embryoid body formation. This study not only demonstrated that sperm and oocytes could be induced from mouse embryonic stem cells (mESCs) in culture, but also implied that DAZL promotes germ cell differentiation (6). In addition, Hübner *et al* (7) demonstrated that mESCs have the potential to develop into oogonia that enter meiosis, recruit adjacent cells to form follicle-like structures, and later develop into blastocysts in culture.

Although previous methods successfully induced mESC differentiation into germ cells *in vitro*, various obstacles remained, including induction inefficiencies and barriers to clinical application associated with controversy regarding embryonic stem cell ethics. In solution to these issues, Evron *et al* (8) reported that human amniotic epithelial cells cultured in medium containing serum substitute supplement could differentiate into oocyte-like cells and express germ cell-specific markers. On the other hand, Dyce *et al* (9) used 5% porcine follicular fluid to induce the differentiation of stem cells derived from porcine epithelial tissue into germ cells. This study revealed that these stem cell-derived epithelial cells were not only able to demonstrate typical oocyte-like structures, but also able to express germ cell markers, such as OCT4, DAZL, VASA and GDF9, after induction by follicular fluid. Also, in a further study, Dyce *et al* (10) induced the differentiation of skin cells from newborn mice into oocyte-like cells using follicular fluid *in vitro*. The results of this study suggest that follicular fluid may contain substances that promote oocyte differentiation and maturation, and that using follicular fluid is a simple and effective method of inducing the differentiation of stem cells into oocyte-like cells. However, if human follicular fluid were to be used to induce the differentiation of human stem cells into oocyte-like cells, issues regarding ethics and identification of appropriate sources of the fluid remain. In view of this, we consider that bovine follicular fluid could be used in place of human follicular fluid to induce the differentiation of human stem cells into oocyte-like cells, just as bovine serum is widely used as a conventional cell culture supplement. Therefore, in the current study, 10% bovine follicular fluid and β-mercaptoethanol were used to induce the differentiation of E-cadherin^+^ human amniotic fluid cells (HuAFCs) into oocyte-like cells. Furthermore, the efficiency of E-cadherin^+^ HuAFC differentiation was evaluated based on oocyte morphology and marker expression.

## Materials and methods

### Isolation and enrichment E-cadherin^+^ cells from HuAFCs using flow cytometry (FCM)

Human amniotic fluids were obtained by ultrasound-guided amniocentesis performed on pregnant females for routine prenatal diagnosis purposes at gestational periods ranging from week 18 to 22. All 5 human samples were obtained following approval from the Ethical Review Board of Shanghai First Maternity and Infant Hospital (Shanghai, China) and after obtaining written informed consent from the subjects. Cells of the E-cadherin^+^ subpopulation were isolated from the human amniotic fluid using 4 μl primary monoclonal antibody [mouse anti-human E-cadherin-fluorescein isothiocyanate (FITC); eBioscience, Inc., San Diego, CA, USA] by storing them at 4°C in phosphate-buffered saline (PBS) for 30 min at a volume of 1.0 ml as described in a previous study (11). Next the cells were washed twice in PBS and plated at a concentration of 1×10^6^ cells/ml in Dulbecco’s modified Eagle’s medium (DMEM):F12 (1:1), supplemented with 10 ng/ml basic fibroblast growth factor (bFGF), 10 ng/ml epidermal growth factor (EGF), 10% fetal bovine serum and 2 mM L-glutamine (Gibco, Gaithersburg, MD, USA). All cells were cultured in a humidified incubator, at 37°C with 5% CO_2_, until 80% confluent. All cells in the present study were cultured in the same conditions until the fourth passage, and then the following experiments were conducted.

### Differentiation into oocyte-like cells

The HuAFCs were differentiated using the methods of previous studies (9,10). Briefly, all HuAFCs were dissociated with 0.125% trypsin-EDTA solution and suspended on Petri dishes with differentiation-inducing cell-conditioned medium [DMEM:F12(1:1) supplemented with 3% KnockOut™ Serum Replacement (Invitrogen Life Technologies, Carlsbad, CA, USA), 0.1 mM β-mercaptoethanol, 10% bovine follicular fluid, 10 ng/ml bone morphogenetic protein (BMP)4, 10 ng/ml bFGF, 10 ng/ml EGF, 1 mM sodium pyruvate, 2 mM L-glutamine, 0.1 mM nonessential amino acids, penicillin (25 U/ml)-streptomycin (925 mg/ml)] The cells were then incubated in a humidified tissue culture incubator containing 5% CO_2_ at 37°C for 15 days until they were completely differentiated as determined in our previous experiments (unpublished data).

### RNA extraction and analysis by quantitative polymerase chain reaction (qPCR)

Total RNA from each cell was isolated with TRIzol reagent (Invitrogen Life Technologies, Grand Island, NY, USA), according to the manufacturer’s instructions. The RNA samples were treated with deoxyribonuclease I (Sigma-Aldrich, St Louis, MO, USA), quantified, and reverse-transcribed into complementary DNA (cDNA) with the ReverTra Ace First Strand cDNA Synthesis kit [Toyobo (Shanghai) Biotech Co., Ltd., Shanghai, China]. qPCR was conducted with a RealPlex4 qPCR Detection system from Eppendorf (Cologne, Germany), with SYBR-Green qPCR Master mix [Toyobo (Shanghai) Biotech Co., Ltd.] as the detection dye. qPCR amplification was performed over 40 cycles with denaturation at 95°C for 15 sec and annealing at 57°C for 45 sec. Target cDNA was quantified with the relative quantification method. A comparative threshold cycle (Ct) was used to determine gene expression relative to a control (calibrator), and steady-state messenger RNA (mRNA) levels were represented as an n-fold difference relative to the calibrator. For each sample, the marker gene Ct values were normalized with the following formula: ΔCt = Ct(genes) - Ct(18S RNA). To determine relative expression levels, the following formula was used: ΔΔCt = ΔCt(sample groups) - ΔCt(control group). The values used to plot relative expression levels of the markers were calculated with the expression 2^−ΔΔCt^. The mRNA levels were calibrated based on the levels of 18S ribosomal RNA. The cDNA of each gene was amplified with primers as described in a former study (8). The primer sequences were as follows: Nanog-F: GATTTGTGGGCCTGAAGAAA; Nanog-R: ATGGAGGAGGGAAGAGGAGA; DAZL-F: CACAGCCTCTGCTCCTCCT; DAZL-R: GCCTCTCTGGAG ATGGTTGA; E-cadherin-F: TTGACGCCGAGAGCTACAC; E-cadherin-R: GACCGGTGCAATCTTCAAA; Dmc1-F: AATCAAATGACTGCCGATCC; Dmc1-R: TTGTTGTTGAAGCATGAGCC; Scp3-F: TCAGAGCCAGAGATTGAAAACA; Scp3-R: TTCATTTTGTGCACCAGTAAGTAGA; Zp3-F: AGTCGTCGTCACACACGGT; Zp3-R: GTACGGACGTCTCAGGATGG; Gdf9-F: ACACTGTTCGGCTCTTCACC; Gdf9-R: TGCGATCCAGGTTAAATAGCA; 18S rRNA-F: CAGCCACCCGAGATTGAGCA; 18S rRNA-R: TAGTAGCGACGGGCGGTGTG.

### Western blot analysis

Cells were lysed using a 2X loading lysis buffer (50 mM Tris-HCl, pH 6.8, 2% sodium dodecyl sulfate, 10% β-mercaptoethanol, 10% glycerol and 0.002% bromophenol blue). The total amount of protein from the cultured cells was subjected to 12% SDS-PAGE and transferred to Hybrid-polyvinylidine fluoride (PVDF) membranes (Millipore, Bedford, MA, USA). Following blocking with 5% (w/v) non-fat dried milk in Tris-buffered saline containing Tween-20 (TBST; 25 mM Tris-HCl, pH 8.0, 125 mM NaCl and 0.05% Tween-20), the PVDF membranes were washed 4 times (15 min each) with TBST at room temperature and incubated with the following primary antibodies: Rabbit anti-human DAZL antibody, Rabbit anti-human E-cadherin antibody, Rabbit anti-human GDF9 antibody, Rabbit anti-human ZP3 antibody, Rabbit anti-human VASA antibody, Rabbit anti-human SCP3 antibody and Rabbit anti-human GAPDH antibody (Santa Cruz Biotechnology Inc., Santa Cruz, CA, USA). Following extensive washing, the membranes were incubated with horseradish peroxidase-conjugated goat anti-rabbit IgG secondary antibody (1:1,000; Santa Cruz Biotechnology Inc.) for 1 h. Subsequent to washing 4 times (15 min each) with TBST at room temperature, the immunoreactivity was visualized by enhanced chemiluminescence using an ECL kit from Perkin-Elmer (Norwalk, CT, USA).

### Immunofluorescent (IF) staining

The cultured cells were washed 3 times with PBS and fixed with 4% paraformaldehyde (Sigma-Aldrich) for 30 min. After blocking, the cells were incubated first with primary antibody overnight at 4°C, and then with FITC- or Cy3-conjugated goat anti-rabbit IgG antibody (1:200; Sigma-Aldrich) and 5 μg/ml DAPI (Sigma-Aldrich) at room temperature for 30 min. Then the cells were thoroughly washed with TBST and viewed through a fluorescence microscope (DMI3000; Leica, Allendale, NJ, USA).

### RNA interference (RNAi) and transfection

The E-cadherin small interfering RNA (siRNA) and negative control (siRNA-mock) was created by Genepharma Co., Ltd. (Shanghai, China) and the transfection method was performed according to the manufacturer’s instructions. Briefly, an E-cadherin-specific siRNA sequence was designed based on the human E-cadherin RNA sequence (Genbank Number: NC_000016.10). The siRNA included a sense strand of 21 nucleotides, a short spacer (TTGATATCCG), the reverse complement of the sense strand, and TTTTT as an RNA polymerase III transcription termination signal. The oligonucleotides used to construct the vectors in this study included 5′-CGGGATCCCAAGATAGGAGTTCTCTGATGCTTGATATCCGGCATCAGAGAACTC CTATCTTTTTTTTCCAAAAGCTTGG-3′ for E-cadherin-siRNA. *Bam*HI and *Hin*dIII sites flanked the siRNA expression cassette and were used to linearize the cassette for ligation into the pRNAT-U6.1/Neo vector for expression of the siRNA in combination with core green fluorescence protein and neomycin as a selectable marker. Plasmids were prepared and purified using an AxyPrep Plasmid Miniprep kit (Axygen Biosciences, Union City, CA, USA) and DNA Gel Extraction kit (Axygen Biosciences) according to the kit instructions. Cells of each group were cultured in a humidified incubator, at 37°C with 5% CO_2_, until 80% confluent. Co-transfection was conducted to transfer 0.3 μg E-cadherin-siRNA or Mock-siRNA plasmids, respectively, with Lipofectamine 2000 reagent (Invitrogen Life Technologies, Carlsbad, CA, USA) according to the product instructions.

### Statistical analysis

Each experiment was performed at least three times and data are shown as the mean ± standard error. The differences were evaluated using Student’s t-test. P<0.05 was considered to indicate a statistically significant difference.

## Results

### E-cadherin^+^ HuAFCs express DAZL highly

**Figure 1:**
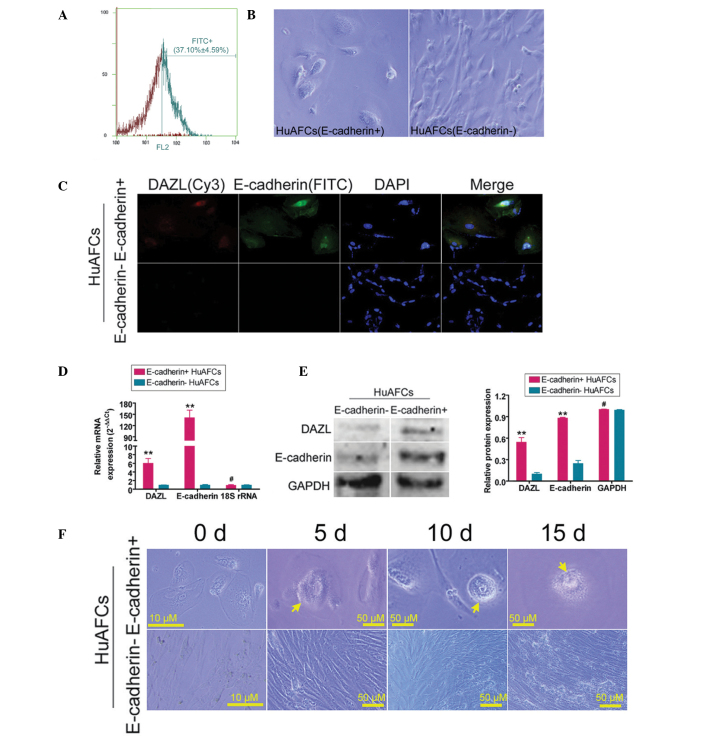
Characterization of E-cadherin^+^ HuAFCs and morphology of oocyte-like cells. (A) Isolation of E-cadherin^+^ HuAFCs from amniotic fluid. The cells were detected by FCM, and E-cadherin^+^ cells represented 37.10±4.59% of the HuAFC population. (B) Morphology of E-cadherin^+^ and E-cadherin^−^ HuAFCs (magnification, ×200). (C) IF staining showing that the levels of DAZL and E-cadherin were elevated in E-cadherin^+^ HuAFCs compared with those in E-cadherin^−^ HuAFCs (magnification, ×200; red fluorescence signal represents the expression of DAZL; green fluorescence signal represents the expression of E-cadherin; blue fluorescence signal represents nuclei with DAPI dye). (D) qPCR analysis of DAZL and E-cadherin mRNA expression in E-cadherin^+^ and E-cadherin^−^ HuAFCs; ^**^P<0.01 vs. E-cadherin^−^ HuAFCs; ^#^P>0.05 vs. E-cadherin^−^ HuAFCs; n=3. (E) Western blot analysis of DAZL and E-cadherin protein expression in E-cadherin^+^ and E-cadherin^−^ HuAFCs; ^**^P<0.01 vs. E-cadherin^−^ HuAFCs; ^#^P>0.05 vs. E-cadherin^−^ HuAFCs; n=3. (F) Day 5 after induction (5 d): Bright field microscopy revealing a marked increase in the volume and size of E-cadherin^+^ HuAFCs, and large round floating cells (diameter >30 μm) were occasionally observed. Day 15 after induction (15 d): Large round floating cells ~50 μm in diameter were detected in E-cadherin^+^ HuAFCs grown in medium containing bovine follicular fluid. A circular ring-banded structure was visible around these cells, which was similar to the zona pellucida of oocytes. Small particulate matter was adhered to one end of a number of the large cells, resembling the polocyte of oocytes. This morphology was not visible in the E-cadherin^−^ HuAFCs. Yellow arrowhead: oocyte-like cells. FITC, fluorescein isothiocyanate; HuAFC, human amniotic fluid cell; FCM, fluorescein isothiocyanate; IF, immunofluorescent; qPCR, quantitative polymerase chain reaction.

A previous study has shown that multiple cell types can be differentiated from HuAFCs (11). However, it was not possible to induce differentiation of all HuAFC sub-populations into oocyte-like cells *in vitro* in the preliminary experiment. In the present study an FCM sorting system was used to isolate and enrich the HuAFC E-cadherin^+^ sub-population. E-cadherin^+^ cells represented 37.10±4.59% of the HuAFCs (Fig. 1A). Microscopically, the E-cadherin^+^ HuAFCs exhibited epithelial cell-like features (‘paving stone’ structure and large nucleus). However, the E-cadherin^−^ HuAFCs exhibited the typical characteristics of fibroblasts (slender, elongated spiral distribution, and small nucleus) (Fig. 1B). To quantify the expression of E-cadherin and DAZL in these sub-populations, qPCR, IF staining and western blotting were used. The expression levels of E-cadherin and DAZL mRNA were significantly higher in the E-cadherin^+^ HuAFCs than those in the E-cadherin^−^ HuAFCs. Western blotting also confirmed that the expression levels of E-cadherin and DAZL (0.884±0.007 and 0.548±0.062, respectively) were significantly higher in the E-cadherin^+^ HuAFCs compared with the levels in the E-cadherin^−^ HuAFCs (0.249±0.040 and 0.102±0.017, respectively). In addition, the IF staining results were consistent with those of the western blotting (Fig. 1C–E). These data indicate that the E-cadherin^+^ HuAFCs expressed DAZL highly.

### E-cadherin^+^ HuAFCs express high levels of germ cell- and oocyte-specific markers

HuAFC sub-populations were induced with medium containing follicular fluid supplement and allowed to differentiate for 15 days. Five days after induction, bright field microscopy revealed that the volume and size of the E-cadherin^+^ HuAFCs had increased significantly, and large round floating cells (diameter >30 μm) were observed occasionally. On day 15, large round floating cells ~50 μm in diameter were detected in the E-cadherin^+^ HuAFCs grown in medium containing bovine follicular fluid. In addition, a circular ring-banded structure closely resembling the zona pellucida of oocytes was observed around these cells. A small particulate matter adhered at one end of a number of the large cells was also observed, which resembled the polocyte of oocytes (Fig. 1F). This feature was not visible in the E-cadherin^−^ HuAFCs. Therefore, the cell morphology analysis revealed that it is possible to induce E-cadherin^+^ HuAFCs to differentiate into oocyte-like cells.

**Figure 2:**
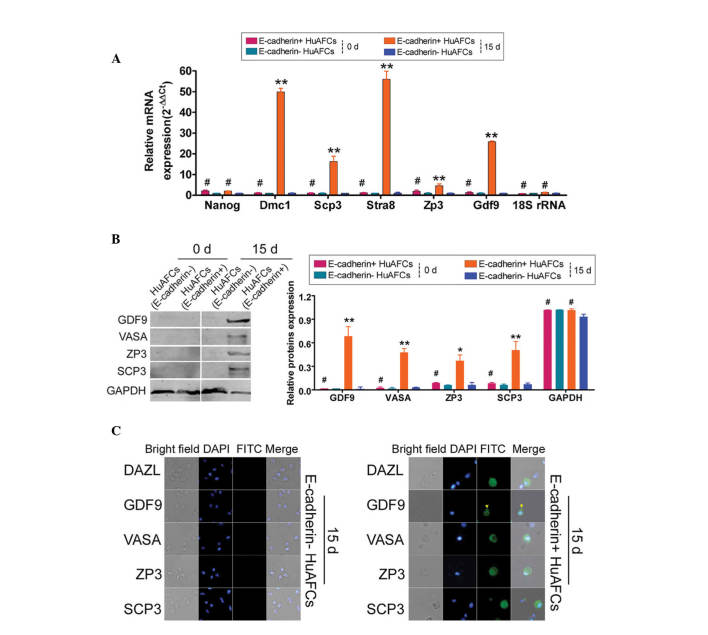
Analysis of marker expression in E-cadherin^+^ HuAFC-derived oocyte-like cells. (A) qPCR analysis of mRNA expression of markers specific for stem cells (NANOG), meiosis (DMC1, SCP3 and STRA8), and oocytes (ZP3 and GDF9) in E-cadherin^+^ and E-cadherin^−^ HuAFCs after induction. On day 15 after induction, the levels of oocyte-specific markers in the E-cadherin^+^ HuAFCs were significantly higher than those in the E-cadherin^−^ HuAFCs. ^**^P<0.01 vs. E-cadherin^−^ HuAFCs; ^#^P>0.05 vs. E-cadherin^−^ HuAFCs; n=3. (B) Western blotting showing that the expression levels of markers specific for meiosis, germ cells and oocytes steadily increased over time in E-cadherin^+^ HuAFCs compared with those in E-cadherin^−^ HuAFCs after induction *in vitro*. ^**^P<0.01 vs. E-cadherin^−^ HuAFCs; ^#^P>0.05 vs. E-cadherin^−^ HuAFCs; n=3. (C) IF staining was performed 15 days after induction to compare the expression levels of oocyte-specific markers in the two sub-populations. The protein expression levels of markers specific for meiosis, germ cells and oocytes were elevated in the E-cadherin^+^ HuAFCs compared with those in the E-cadherin^−^ HuAFCs (magnification, ×200). Yellow arrowhead: Small particulate matter adhered to one end of large cell, resembling the polocyte of oocytes. HuAFC, human amniotic fluid cell; FITC, fluorescein isothiocyanate; qPCR, quantitative polymerase chain reaction; IF, immunofluorescent.

qPCR and western blotting were performed to quantify the expression of markers specific for meiosis, germ cells and oocytes in the sub-populations. The qPCR and western blotting revealed that there were high expression levels of NANOG (a stem cell marker) in the sub-populations on day 0 compared with those of the other markers. No significant differences in the expression levels of NANOG between the two sub-populations were identified, and the two groups did not express markers specific for meiosis (DMC1, SCP3 and STRA8) and oocytes (ZP3 and GDF9) on day 0. However, following induction of differentiation, the expression levels of markers specific for meiosis (DMC1, SCP3 and STRA8), germ cells (DAZL and VASA), and oocytes (ZP3 and GDF9) increased between days 0 and 15 in the E-cadherin^+^ HuAFCs compared with those in the E-cadherin^−^ HuAFCs, in which levels did not increase. On day 15 after induction, the levels of these markers in the E-cadherin^+^ HuAFCs were significantly higher than those in the E-cadherin^−^ HuAFCs (Fig. 2A and B). IF staining was performed 15 days after induction to compare the expression levels of markers specific for stem cells, meiosis, germ cells and oocytes in the two sub-populations (Fig. 2C). The protein expression levels of the markers specific for meiosis, germ cells, and oocytes were elevated in the E-cadherin^+^ HuAFCs compared with those in the E-cadherin^−^ HuAFCs. These results indicated that the oocyte phenotype of the E-cadherin^+^ HuAFCs was clearer than that of the E-cadherin^−^ HuAFCs after induction, and the E-cadherin^+^ HuAFCs expressed high levels of markers specific for germ cells and oocytes following induction.

### E-cadherin RNAi expression influences E-cadherin^+^ HuAFC differentiation into oocyte-like cells

**Figure 3:**
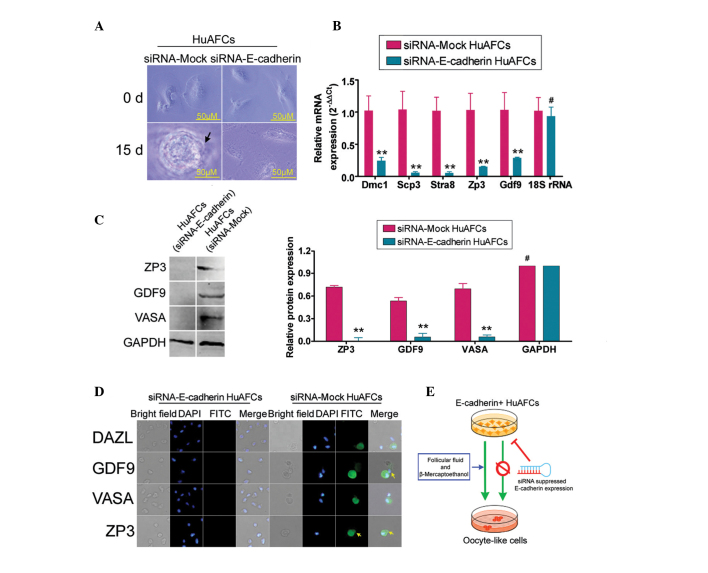
Suppression of E-cadherin expression by siRNA influenced E-cadherin^+^ HuAFC differentiation into oocyte-like cells. (A) Cell morphology prior to and following induction of siRNA-E-cadherin HuAFCs and siRNA-mock HuAFCs. On day 15 after induction, the siRNA-mock cells appeared to be banded by a circular structure closely resembling the zona pellucida of oocytes. This feature was not visible in the siRNA-E-cadherin HuAFCs. Black arrow: Polocyte of oocytes. (B) qPCR showing that the levels of oocyte-specific markers in the siRNA-E-cadherin HuAFCs were significantly lower than those in the siRNA-mock HuAFCs 15 days after induction. ^**^P<0.01 vs. siRNA-mock HuAFCs; ^#^P>0.05 vs. siRNA-mock HuAFCs; n=3. (C) Western blotting showing that ZP3, GDF9 and VASA expression levels decreased over time in siRNA-E-cadherin HuAFCs compared with those in siRNA-mock HuAFCs 15 days after induction *in vitro*. ^**^P<0.01 vs. siRNA-mock HuAFCs; ^#^P>0.05 vs. siRNA-mock HuAFCs; n = 3. (D) IF staining was performed 15 days after induction to compare the expression levels of oocyte-specific markers in the two sub-populations. The protein expression levels of DAZL, GDF9, VASA and ZP3 were reduced in the siRNA-E-cadherin HuAFCs compared with those in the siRNA-mock HuAFCs (magnification, ×200). Yellow arrow: Polocyte of oocytes. (E) Using bovine follicular fluid, E-cadherin^+^ epithelial HuAFCs could be induced to differentiate into oocyte-like cells expressing markers specific for germ cells and oocytes. Suppression of cadherin by siRNA influenced E-cadherin^+^ HuAFC differentiation into oocyte-like cells. siRNA, small interfering RNA; HuAFC, human amniotic fluid cell; FITC, fluorescein isothiocyanate; qPCR, quantitative polymerase chain reaction; IF, immunofluorescent.

qPCR, western blotting and IF staining demonstrated that the expression levels of markers specific for meiosis (DMC1, SCP3 and STRA8), germ cells (DAZL and VASA), and oocytes (ZP3 and GDF9) were markedly lower in the siRNA-E-cadherin HuAFCs than those in the siRNA-mock HuAFCs after induction. The oocyte phenotype of the siRNA-E-cadherin HuAFCs was also less clear than that of the siRNA-mock HuAFCs after induction (Fig. 3). These results indicate that the efficiency of oocyte-like cell induction was reduced following E-cadherin downregulation in HuAFCs.

## Discussion

The successful induction of stem cell differentiation into germ cells *in vitro* has important implications for reproductive regenerative medicine and tissue engineering. However, the complex microenvironment (niche) of germ cell development *in vitro* and its relation to meiosis increase the difficulty of successful induction *in vitro*. Although the induction of embryonic stem cell, induced pluripotent stem (iPS) cell and germ stem cell differentiation into sperm/oocyte-like cells has been successful (1–3,5,7), the efficiency of induction was low and the process was complex. Generally, the choice of seed cells and induction culture system has great impact on the efficiency of differentiation. Evron *et al* (8) found that human amniotic epithelial cells, possessing the characteristics of epithelial cells, could be induced to differentiate into oocyte-like cells when cultured in medium containing serum substitute supplement, and could express markers specific for germ cells and oocytes. Taking into account that germ cells can be derived from the embryonic ectoderm, it may be possible to induce differentiation of stem cells with epithelial cell characteristics into germ-like cells more easily *in vitro*. Currently, the use of stem cells (embryonic stem cells, iPS cells and germ cells) faces multiple difficulties including ethical restrictions, induction inefficiencies, complex culture requirements and limited sources (3,6,7). Thus, stem cells are not ideal seed cells. Although human amniotic epithelial cells are good seed cells, possessing embryonic stem cell pluripotency and low immunogenicity characteristics, the necessary complex primary isolation and culture procedures used for them have led to difficulties in their application (6). HuAFCs are currently used for routine prenatal genetic diagnosis of a wide range of fetal abnormalities. However, amniotic fluid contains numerous cell types derived from the developing fetus. Previous studies have demonstrated the ability of HuAFCs to differentiate into ectodermal, endodermal, mesodermal, hepatic and cardiac muscle cells (11–14). Therefore, given their embryonic stem cell pluripotency characteristics, HuAFCs can differentiate into all three germ layers. Additionally, previous studies have indicated that HuAFCs express a variety of growth factors, including EGF, bFGF, transforming growth factor α and β, BMP 4, and the stem cell markers NANOG and nestin (11,14). More importantly, HuAFCs lack major histocompatibility complex class II antigens and express only low levels of major histocompatibility complex class I antigens (11–14). Previous studies have reported that the dopaminergic neuron-like cells derived from HuAFCs ameliorated behavioral recovery in a rat model of Parkinson’s disease (12), and that HuAFCs could be induced to differentiate into pancreatic β-cell-like cells (14). HuAFCs are more easily obtained than other adult stem cells, making them a potential autologous donor source for stem cell therapy. On the other hand, the niche of germ cell development and maturation *in vivo* is complex. A number of different hormones and cytokines can be added into culture medium to induce stem cell differentiation into germ cells *in vitro* (3,8–10). Consequently, not only do experimental expenses and uncertainty increase, the introduction of excess exogenous proteins is accompanied by the risk of failure of future transplantation treatment *in vivo*. In addition, it is difficult to quantify the hormone and cytokine concentrations used to induce germ cells, which renders induction inefficient. Dyce *et al* (9,10) demonstrated that adding 5% porcine follicular fluid to conventional medium induced skin stem cells to express DAZL, GDF9 and VASA successfully, and these cells possessed the typical follicular cell structure. The results of these studies by Dyce *et al* reveal that instead of a series of hormones and cytokines, a particular amount of follicular fluid can be used to induce stem cell differentiation into oocyte-like cells, which is more convenient and efficient.

Theoretically, it is more feasible to use human follicular fluid to induce HuAFC differentiation into oocyte-like cells, as it would avoid contamination from heterogeneous proteins. However, human follicular fluid is obtained by ovarian hyperstimulation of females who intend to undergo *in vitro* fertilization. Therefore, ethical restrictions on the use of human follicular fluid remain. However, under normal circumstances, bovine serum has proven sufficiently stable to maintain the growth of a variety of types of human cell *in vitro* (8). Thus, bovine follicular fluid was used in the present study as an additive to induce HuAFC differentiation into oocyte-like cells *in vitro*, a process without ethical constraints and that had low toxicity. In the current study, FCM sorting technology was used to isolate and enrich an E-cadherin^+^ HuAFC sub-population. Using this biomarker and method, epithelial cells were sorted from the HuAFCs. Next, bovine follicular fluid was added to the basic medium, which was used to induce E-cadherin^+^ HuAFC differentiation into oocyte-like cells *in vitro*. Molecular biology techniques indicated that the E-cadherin^+^ HuAFCs, but not the E-cadherin^−^ sub-population, highly expressed DAZL. It has been demonstrated that DAZL is a germ cell-specific gene that exhibits critical roles in germ cell development and differentiation (6). Furthermore, DAZL is involved in the translational regulation of MVH and SCP3 in meiotic cells, and has been demonstrated to be a key intrinsic factor in the initiation of meiosis in response to extrinsic signaling (6). The E-cadherin^+^ HuAFCs in the present study highly expressed DAZL protein, revealing that it may be easier to induce these cells to differentiate into oocyte-like cells. To verify this hypothesis, the E-cadherin^+^ and the E-cadherin^−^ HuAFCs were induced under the same conditions. Subsequently, morphological and molecular identification indicated that not only oocyte morphology, but also biomarkers specific for germ cells and oocytes could be found in the E-cadherin^+^ HuAFCs, but not the E-cadherin^−^ HuAFCs, following induction. The results of the present study reveal that bovine follicular fluid induces E-cadherin^+^ HuAFCs to express oocyte-specific proteins and establish the oocyte phenotype. The efficiency of oocyte-like cell differentiation in the E-cadherin^+^ HuAFCs was then determined when the expression of the E-cadherin gene was silenced by being specifically targeted by siRNA. The results displayed that the expression of DAZL also decreased after E-cadherin downregulation, indicating an association between the two. In addition, the expression of the oocyte and germ cell-specific markers in the E-cadherin^+^ HuAFCs was significantly downregulated when RNAi was used to silence the E-cadherin gene.

In conclusion, the present study successfully established an oocyte-like cell differentiation system *in vitro*. Bovine follicular fluid induced E-cadherin^+^ HuAFC differentiation into oocyte-like cells that expressed markers specific for germ cells and oocytes. In addition, it was found that the E-cadherin^+^ HuAFCs exhibited the potential characteristics of DAZL protein expression. We hypothesize that bovine follicular fluid acts on DAZL protein and promotes E-cadherin^+^ HuAFCs to differentiate into oocyte-like cells.

## Abbreviations

DAZL: deleted in azoospermia-like protein
DMC1: disrupted meiotic cDNA
FCM: flow cytometry
GDF9: growth differentiating factor 9
HuAFCs: human amniotic fluid cells
IVF: *in vitro* fertilization
mESCs: mouse embryonic stem cells
MVH: mouse VASA homolog
OCT4: octamer-binding transcription factor 4
RNAi: RNA interference
SCP3: synaptonemal complex protein 3
siRNA: small interfering RNA
STRA8: stimulated by retinoic acid 8
ZP3: zona pellucida 3 protein
